# Editorial: Light Regulation of Metabolic Networks in Microbes

**DOI:** 10.3389/fmicb.2022.829106

**Published:** 2022-02-07

**Authors:** Monika Schmoll, Catalina Sanz, Weiwen Zhang

**Affiliations:** ^1^AIT Austrian Institute of Technology GmbH, Center for Health and Bioresources, Tulln, Austria; ^2^Department of Microbiology and Genetics, University of Salamanca, Salamanca, Spain; ^3^Laboratory of Synthetic Microbiology, School of Chemical Engineering and Technology, Tianjin University, Tianjin, China

**Keywords:** light response, circadian rhythm, photoreceptor, substrate utilization, phenotypic plasticity, phenotype microarrays, RNAseq

Day and night dominate our life and profoundly influence society. We feel the importance of the differences between day and night if we need sleep or if we have a jetlag. Both phenomena are triggered by our circadian clock, which can be influenced by light. Deprivation of light as well as perturbation of the circadian clock leads to severe health problems (Lee et al., 2021).

For many people, including researchers, it comes as a surprise that microbes also react strongly to light and that they have sophisticated circadian clocks as well (Hurley et al., 2016a; Alsanius et al., 2019; Corrochano, 2019). Metabolism of fungi is subject to light response (Tisch and Schmoll, 2010; Hurley et al., 2016b). Growth in the dark is associated with fundamentally different gene regulation patterns than growth in light in fungi (Chen et al., 2009; Stappler et al., 2017a). Rhythmically cycling gene expression following the biological clock is not limited to clock genes or photoreceptors. Numerous genes involved in metabolic functions show circadian regulation as well (Hurley et al., 2014; Diaz and Larrondo, 2020). In recent years, light response of non-phototrophic bacteria has also been receiving increased attention and also there, metabolic alterations were detected (Gharaie et al., 2017). With respect to natural habitats, light pollution—also known as artificial light at night (ALAN)—even impacts microbial community composition (Hölker et al., 2015). Reproduction processes, for example sexual development of fungi, are dependent on light and darkness (Debuchy et al., 2010).

However, despite the wealth of data already available, the question remains why growth in light on the same medium and under equal conditions alters the metabolism and physiology of fungi so drastically, as seen in the case of the fungus *Trichoderma reesei* (Stappler et al., 2017b).

The articles of this Research Topic highlight effects of light on fungi as well as non-phototropic bacteria, reflecting the breadth of studies on light response in microbes. Their findings bring us one step closer to understanding the biological relevance behind the complex regulation of light dependent processes in microbes.

For bacteria, the studies in this Research Topic tackle the issue of light response from two different angles: substrate utilization and growth as well as genome wide transcriptome alterations. Moreover, both studies showed that in the microbes responding to light putative photoreceptor proteins were also detected.

Alsanius et al. investigated the influence of white, blue, and red light on non-phototrophic bacteria inhabiting the phyllosphere. They found altered substrate utilization patterns for carbon, nitrogen, and phosphorous sources in dependence of light. These alterations varied for different bacteria and the extent of the responses was strain specific. Interestingly, biofilm formation and biosurfactant production also correlate with responses to light, although in this case it remains to be shown whether light directly influences these phenomena or if the effects are indirect *via* an alteration of growth rates due to illumination. Overall, this study nicely shows that light responses in bacteria are species specific and it indicates that the microbiome of the phyllosphere likely changes in different light regimes.

Hempel et al. investigated transcriptomes of two species of actinobacteria over a time course of several hours. They especially looked at genes involved in carbon metabolism. This study shows interesting differences in the regulation of genes associated with the TCA cycle between species. Interestingly, the genes responding to light in these two species are largely dissimilar, even though many light-dependent effects are comparable. The authors conclude that the reaction to light is widespread, but direction and specificities are different, suggesting that different species deal with light in unique ways.

The two studies on fungal light responses tackle the question of how the light response machinery works, with intriguing investigations of fungal photoreceptors in *Fusarium fujikuroi* and *Trichoderma atroviride*. Pardo-Medina et al. focused on the white collar-1 homolog WcoA of *F. fujikuroi* and could show that most of the fast, intermediate, and slow responses to light in terms of gene regulation are dependent on WcoA. The impact of light was especially significant for the regulation of secondary metabolite gene clusters. Moreover, reminiscent of earlier studies in *T. reesei* (Tisch and Schmoll, 2013), they also showed that *F. fujikuroi* WcoA has broad functions in darkness—to an even larger extent than *T. reesei*. Consequently, the previously revealed flat hierarchy of the transcription factor function in *Neurospora crassa* (Smith et al., 2010) is likely to be a more general phenomenon.

Pola-Sánchez et al. added a new and important chapter to the function of the photoreceptor ENV1: the *Trichoderma* homolog of *N. crassa* VVD. This PAS-domain protein already showed diverse functions in *T. reesei* (Schmoll, 2018) and adds intriguing complexity to the photoperception machinery. Pola-Sánchez et al. now connect the photoreceptor function of ENV1 to DNA repair and central metabolism in *T. atroviride* and postulate that ENV1 prevents exacerbated light responses. In *N. crassa* a comparable function was shown in that VVD blocked excitability of the light response machinery after a first light pulse (Schwerdtfeger and Linden, 2001, 2003). Pola-Sánchez et al., further show that the cryptochrome/photolyase family proteins CRY1 and CRY-DASH contribute to regulation of conidiation, and that ENV1 as well as CRY1 impact light dependent carbon and nitrogen metabolism.

In conclusion, both fungi and bacteria show distinct responses to light. As could be concluded from previous work on microbial light response, the studies here also reflect that the aim of metabolic responses for an optimal coping with light is similar for different species. However, the mechanisms for achieving this aim in terms of gene expression, regulation, and substrate utilization are divergent. Consequently, for a given species, prediction of how it might react to light physiologically is difficult. While the studies presented in this Research Topic provide intriguing new insights into microbial light response, still more investigations are needed to understand the complexity of light response in microbes. Moreover, together with previous work, these studies highlight the importance of using controlled light conditions when working with microbes, as there is hardly any regulatory aspect of microbial physiology that is known to not be subject to alteration by light conditions.

## Author Contributions

MS drafted the manuscript and wrote the final version of the manuscript. CS and WZ edited the draft. All authors read and agreed to submission of the article. All authors contributed to the article and approved the submitted version.

## Conflict of Interest

Author MS was employed by the company AIT Austrian Institute of Technology GmbH. The remaining authors declare that the research was conducted in the absence of any commercial or financial relationships that could be construed as a potential conflict of interest.

## Publisher's Note

All claims expressed in this article are solely those of the authors and do not necessarily represent those of their affiliated organizations, or those of the publisher, the editors and the reviewers. Any product that may be evaluated in this article, or claim that may be made by its manufacturer, is not guaranteed or endorsed by the publisher.
